# Conception and implementation of a certification system for quality control of cochlear implant treatment in Germany

**DOI:** 10.1007/s00106-023-01306-w

**Published:** 2023-06-12

**Authors:** T. Stöver, S. K. Plontke, O. Guntinas-Lichius, H.-J. Welkoborsky, T. Zahnert, K. W. Delank, T. Deitmer, D. Esser, A. Dietz, A. Wienke, A. Loth, S. Dazert

**Affiliations:** 1 Department of Otorhinolaryngology, Frankfurt University Hospital, Theodor-Stern-Kai 7, 60590 Frankfurt, Germany; 2 Department of Otorhinolaryngology, Head and Neck Surgery, University Medical Center Halle, Halle (Saale), Germany; 3 Department of Otorhinolaryngology, Jena University Hospital, Jena, Germany; 4 Hospital for Otorhinolaryngology, Klinikum Nordstadt, Hanover, Germany; 5 Department of Otorhinolaryngology, Dresden University Hospital, Dresden, Germany; 6 Department of Otorhinolaryngology, Ludwigshafen Hospital, Ludwigshafen, Germany; 7 Department of Otorhinolaryngology, Leipzig University Hospital, Leipzig, Germany; 8 Law office WBK, Cologne, Germany; 9 Department of Otorhinolaryngology, University Hospital (St. Elisabeth Hospital), Bochum, Germany

**Keywords:** Rehabilitation, Implantable neurostimulators, Clinical practice guidelines, Prostheses and implants, Quality control

## Abstract

A standardized and structured process is indispensable for optimal hearing rehabilitation with cochlear implants (CI). The Executive Committee of the German Society of Otorhinolaryngology, Head and Neck Surgery (DGHNO-KHC), initiated the conception of a certification program and a Whitepaper based on the Association of the Scientific Medical Societies in Germany (AWMF) clinical practice guideline (CPG) describing the current medical standards of CI care in Germany. The goal was to independently confirm implementation of this CPG and make this information publicly available. With the support of an independent certification organization, successful implementation of the CI-CPG by a hospital would then be verified and confirmed by awarding the “Cochlear implant-provision institution” (*Cochlea-Implantat-versorgende Einrichtung*, CIVE) quality certificate. A structure for implementation of a certification system was developed based on the CI-CPG. The following steps were required: 1) conception of a quality control system for certification of hospitals working in accordance with the CI-CPG; 2) development of required structures for an independent review of quality-relevant structure, process, and result parameters; 3) development of a standard procedure for independent certification of hospitals; 4) development of a certificate and a logo to demonstrate successful certification; 5) practical implementation of the certification. Following design of the certification program and the required organizational structure, the certification system was successfully launched in 2021. Applications for the quality certificate could be formally submitted from September 2021. A total of 51 off-site evaluations were performed by December 2022. In the first 16 months from introduction, 47 hospitals were successfully certified as CIVE. In this period, 20 experts were trained as auditors, who have since then carried out 18 on-site audits in hospitals. In summary, the conceptual design, structure, and practical implementation of a certification program for quality control in CI care was successfully implemented in Germany.

**Supplementary Information:**

The online version of this paper (10.1007/s00106-023-01306-w) contains supplementary material to Table 1: “Survey and key data sheet for initial certification as cochlear implant (CI) provision institution (CIVE),” which is available to authorized users.

## Relevance of CI care process

Providing patients who suffer from severe hearing loss or deafness with a cochlear implant (CI) has been the “gold standard” in hearing rehabilitation of affected patients for many years [1]. CI treatment is a very successful, but also complex, time-consuming, cost-intensive, and lifelong care process that is the responsibility of an otorhinolaryngology specialist and is not limited to the surgical implantation of the CI alone. Against this background, both an multidisciplinary approach and the control of the individual diagnostic, therapeutic, rehabilitative, and quality aspects are essential in order to provide patients with effective and safe care.

Although the success of CI therapy is undisputed and has been proven beyond doubt for more than 35 years by a large number of scientific publications, there is nevertheless a considerable range of variation with regard to the results achieved in individual cases. The results are significantly influenced by the individual factors of a patient, such as the duration of deafness or residual hearing. In addition, outcomes are also determined by surgical technique, audio processor fitting, rehabilitation, and follow-up [2]. Patients with hearing loss require interdisciplinary care, that includes audiological, hearing and speech therapeutic, pediatric audiological, educational, and socio-medical aspects.

Optimal use of the CI therefore requires lifelong care of implant-treated patients. This applies both to children and adults. A standardized and structured care process is the prerequisite for the best possible and lifelong treatment success. Another goal of this structuring is to minimize the risk of insufficient quality of outcome, such as inadequate hearing and speech development, lack of or failure to regain participation in daily life, and medical complications. Also, an insufficiently treated hearing and speech impairment often leads to a significant impairment of quality of life [3].

## Medical standards

Medical standards are usually defined with the help of clinical practice guidelines (CPG) and established at a national level. In Germany, this is done in a standardized procedure via the Association of Medical Scientific Societies (*Arbeitsgemeinschaft der Wissenschaftlichen Medizinischen Fachgesellschaften e.* *V.*, AWMF). In October 2020, the new version of the CI CPG was prepared under the leadership of the German Society of Otolaryngology, Head and Neck Surgery (DGHNO-KHC). Since then, this guideline represents the standard of CI treatment applicable in Germany (AWMF Register No. 017-071; [4]).

For the first time, this CI CPG covers essential aspects of quality control with regard to structural quality, process quality, and quality of results in CI care in Germany. Examples of this include the spatial and technical equipment, the qualification of personnel, the minimum number of staff, the guarantee of the fitting and rehabilitation process, as well as the guarantee of lifelong aftercare for patients at a facility. This guideline thus represents a milestone in the quality control of CI care in Germany.

The content of a CPG corresponds to the current state of scientific knowledge. The verification of compliance with the guideline content has not been sufficiently possible to date. On the one hand, there are clear recommendations for the structural, process, and outcome quality of CI care in Germany, and on the other hand, there has been no objective proof of the implementation of the guideline content. This gap should be closed by the introduction of the certification program described here. With the involvement of an independent and accredited certification organization with experience in the healthcare sector, the intention was to certify the successful implementation of the guideline content by awarding the quality certificate “Cochlear implant-provision institution” (*Cochlea-Implantat versorgende Einrichtung, *CIVE) to qualified hospitals.

On the initiative of the executive committee of the DGHNO-KHC, a Germany-wide quality control program should therefore be introduced that identifies which facilities implement the currently valid recommendations of the CPG. For this purpose, the following goals should be achieved:

1. Conception of a quality control system for the certification of hospitals working according to guideline
2. Development of the necessary structures for independent verification of quality-relevant structural, process, and outcome parameters
3. Development of a standard procedure for the independent certification of hospitals
4. Development of a certificate and a logo to prove successful certification
5. Practical implementation of the certification program

## Material and methods

### Development of the medical–scientific basis for CI certification

In Germany, the preparation of a medical–scientific-founded CPG is based on a standardized process of the AWMF. This process results in a consensus CPG. This guideline is then regarded as the uniform recommendation for diagnostics, therapy, and aftercare for all disciplines working in this field and it applies throughout Germany.

The DGHNO-KHC was commissioned by the AWMF as the “lead” society for the development of the CI CPG. After the first version of the CI CPG was established in 2001, the current third version was consented and published in October 2020 after a strictly standardized development process [4]. The current CPG was developed in close cooperation with all other medical and scientific societies, in particular the German Society for Phoniatrics and Pediatric Audiology (DGPP) and the German Audiological Society (DGA).

On the basis of this guideline, the “White Paper on Cochlear Implant Care in Germany” (CI White Paper) was developed under the leadership of the DGHNO-KHC. The CI White Paper represents a practical instruction for the transfer of the CPG content into clinical practice. The CI White Paper was also technically consulted with the DGPP and the DGA and adopted and published by the executive committee of the DGHNO-KHC in May 2021 [5].

### Key content of the CI CPG and the CI White Paper in Germany

**Fig. 1:**
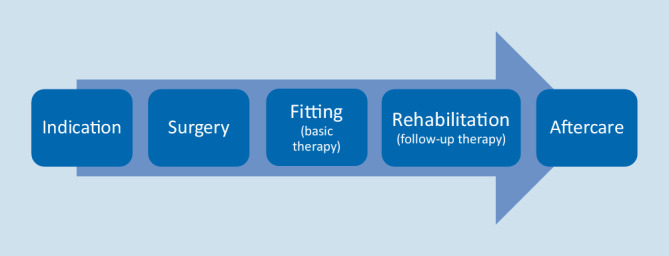
Schematic representation of the process steps required for cochlear implant (CI) care according to the CI guideline

The content-related goal of the CI CPG and thus of the CI White Paper is the description of relevant quality criteria of a scientifically based CI care. This includes not only implantation, but also the entire care process from indication, surgery, fitting (basic therapy), rehabilitation (follow-up therapy) and aftercare (Fig. 1). To this end, the fulfillment of the structure-, process-, and outcome-relevant quality parameters described in the CPG and the CI White Paper is the indispensable prerequisite for a successful hearing rehabilitation of patients with CIs. These parameters subsequently form the basis for the successful certification of a facility.

The care of a patient with a CI is an interdisciplinary process with the close interaction of different disciplines, e.g., ENT physicians, phoniatrists/pediatric audiologists, audiologists, neuroradiologists, anesthesiologists, pediatricians, pedagogues, speech therapists, and others. The final responsibility for this process is undoubtedly based on medical aspects (indication and surgical implantation) and also on legal requirements in the hands of the ENT specialist surgeon or at the institution where a patient receives the CI (= “operator of the implant” according to the German *Medizinprodukte-Betreiberverordnung, *MPBetreibV; [6]). In Germany, these facilities are usually hospitals, i.e., departments of otorhinolaryngology–head and neck surgery, as only these provide all the necessary structural features to be able to fully implement the care process outlined in the CPG and the White Paper. Therefore, only appropriately equipped and structured hospitals for otorhinolaryngology–head and neck surgery are currently eligible to apply for a CI certificate. In order to enable a differentiation of corresponding hospitals, the term “Cochlear implant-provision institution” (*Cochlea-Implantat versorgende Einrichtung, *CIVE) was introduced in Germany. This was necessary because to date there was no uniform use of other terms (e.g., “CI center,” “CI hospital”, etc.) that were linked to the fulfillment of quality requirements. By using the term “CIVE,” the certified facility provides evidence of meeting the quality parameters required by the CPG and the CI White Paper. This means that a hospital using the CIVE designation must have been successfully certified in accordance with the professionally and scientifically defined requirements.

### Development of the content-related basis and objectives of the certification

On the basis of the CI CPG and the CI White Paper, a questionnaire had to be developed that would elicit the essential requirements from both documents. This was necessary because the CPG comprises a total of 78 pages of text. Finally, a catalog comprising 35 questions/items was extracted from this document. This survey and key data sheet thus compiles the essential parameters of the structural, process, and outcome quality of the CI care process. The information is based on a “yes or no” answer and confirmation of structural specifications, such as qualification and number of staff. Another essential element is the assurance of the individual process steps of CI care, such as fitting the audio processor, ensuring rehabilitation, and lifelong follow-up. The survey and key data sheet is the basis of the certification process, which is verified by an independent certification organization.

### Formal basis of the establishment of certification

After passing a resolution in 2017, the executive committee of the DGHNO-KHC established a task force to further develop the topic of quality control in CI care in Germany. The work presented had a lead-in of several years and represents the product of intensive conceptual work. The technical leadership with regard to the medical–scientific content of the certification lay with the presidium of the DGHNO-KHC.

### Structural basis of certification

In order to ensure an independent review of the quality criteria according to the applicable CPG, an independent certification organization (ClarCert GmbH, Neu-Ulm, Germany), experienced and accredited in the healthcare sector, was commissioned by the DGHNO-KHC to ensure the technical implementation of the certification process.

### Certification procedure

**Fig. 2:**
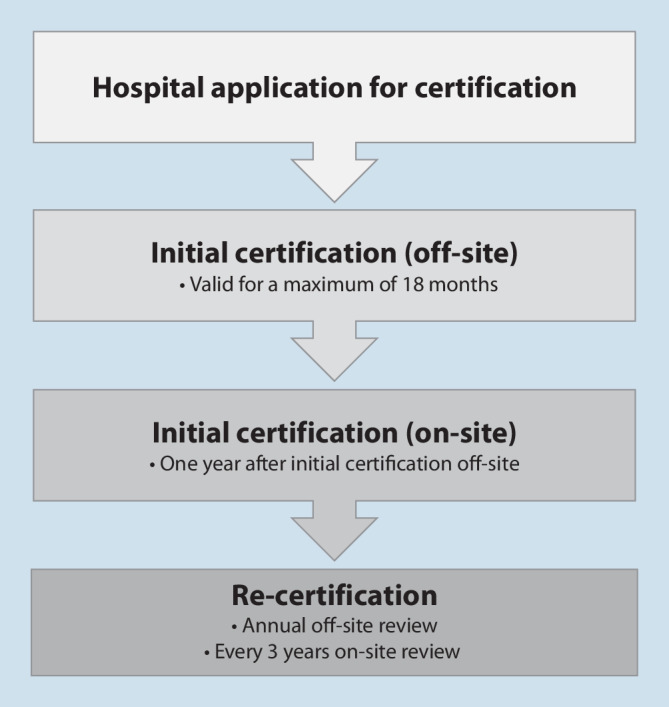
Presentation of the individual steps for obtaining and maintaining the certificate “Cochlear Implant Provision Institution” (*Cochlea-Implantat versorgende Einrichtung*, CIVE). Also shown are the re-certification intervals (once a year off-site examination and every 3 years on-site)

The certification process is subject to a standardized procedure (Fig. 2). The process begins with the application of the facility to the certification organization. This application includes the provision of information critical to certification for the assessment of quality-relevant structural, process, and outcome aspects. This information is provided in writing by the facility to be certified based on the survey and key data sheet. The initial certification is based on the evaluation of the survey and key data sheet submitted in writing (“off-site audit”). If the necessary requirements are met, the certificate is initially granted for a period of 18 months. The certificate can be applied for and awarded for CI care for children *or* adults, but also for children *and* adults if the requirements are met.

The survey and key data sheet is provided by the CIVE once a year after initial certification in order to identify possible structural changes (e.g., personnel changes) and take them into account for re-certification.

Beginning with the first year after initial certification, an on-site audit is performed by trained technical experts. The task of the technical experts is to verify on-site the information provided by the CIVE in the survey and key data sheet. In addition, potential for improvement is to be constructively identified and, if necessary, deviations from the quality standards defined in the CPG are to be documented. The report of the technical expert results in a recommendation for awarding the certificate. An on-site audit by the technical expert is carried out at 3‑year intervals to maintain the certificate.

### Organizational structures of the certification process

**Fig. 3:**
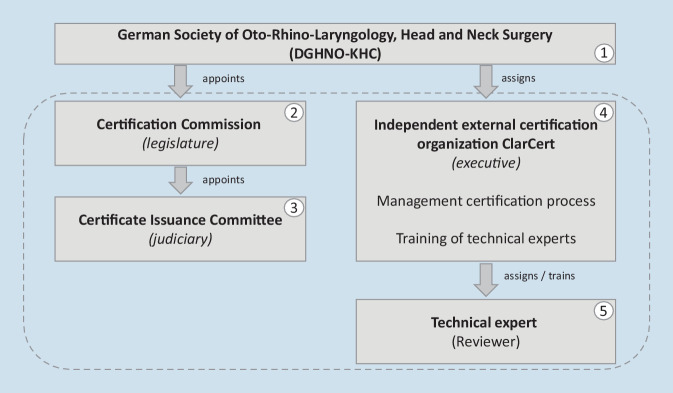
Presentation of the organizational chart of the responsible acting organizational structures of the certification process

To ensure a transparent approach to the certification process, there was a need to establish organizational structures with clearly assigned responsibilities (Fig. 3). In detail, these were:

- German Society for Otorhinolaryngology, Head and Neck Surgery
- Certification commission
- Certificate issuance committee
- Independent external certification organization
- Technical experts

#### German Society for Otorhinolaryngology, Head and Neck Surgery (DGHNO-KHC)

In consensus with other professional societies active in the field of CI care (especially the DGPP and the DGA), the national medical–scientific professional society (AWMF) developed the basis for the currently valid CI CPG in Germany. On the basis of the CPG, a practical recommendation for the implementation of the CPG was developed (CI White Paper). The professional responsibility for the content of the care process ultimately lay with the DGHNO-KHC.

#### Certification commission

The DGHNO-KHC appoints the members of a certification commission, which has the function of the “legislature” in the certification program. The task of the commission is to develop the content of the survey and key data sheet on the basis of the CI CPG and the CI White Paper. The survey and key data sheet contains the essential quality aspects of the CPG and thus represents the basis for reviewing the structural, process, and outcome quality of a CIVE.

#### Certificate issuance committee

The “certificate issuance committee” performs the task of the “judiciary” in the certification program, i.e., the assessment of the applications submitted. This working level is composed of technical experts appointed by the “certification commission,” who decide on the acceptance or rejection of the applications for certification, as well as on the ability of hospitals to apply for the certification program.

#### Independent external certification organization

The tasks of the independent certification organization (the company ClarCert) correspond to the “executive” domain. This includes the technical preparation of decisions on certification applications, verification of formal eligibility to apply, verification of fulfillment of the formal structural, process, and outcome requirements (survey and key data sheet), and qualification of the technical experts to conduct the on-site audits. The organization also independently monitors the formal steps of the certification and the content-related fulfillment of the quality criteria specified by the certification commission for the processes of the certification program.

#### Technical experts

The task of the “technical experts” is to conduct the on-site audits, to verify the information previously provided in writing by the applicant facility, and to identify potential for improvement or deviations in the facilities. Otorhinolaryngology specialists with experience in the field of CI care could apply to the certification organization for this activity. The corresponding training courses were conducted by the independent certification organization in order to qualify the technical experts professionally and formally for their activities as reviewers. The successfully qualified technical experts are then commissioned by the certification organization in the course of the certification procedure to carry out the on-site audit at the applicant facility. Here, the technical experts prepare an audit report and make a recommendation on whether to award the certificate. A rejection or an acceptance under defined conditions can be recommended by the technical experts. The recommendation of the technical experts is the basis for the decision of the certificate issuance committee, which finally decides on the issuance of a certificate.

### Certificate and logo

After successful initial certification or successful re-certification through an on-site audit, the certificate confirms that the applicant facility is a “Cochlear implant-provision institution” (*Cochlea-Implantat versorgende Einrichtung*; CIVE). The certificate is delivered by the certification organization, but the executive committee of DGHNO-KHC e. V. is responsible for the content and signs it. The certificate is issued for CI care of children, of adults or of children and adults. The written certificate can be used in the hospital as well as in external communication. Authorization to use the certification logo is awarded for the duration of the respective certificate validity (18 months or 3 years). The logo can also be used by the successfully certified facility for internal and external communication.

## Results

The establishment of the described organizational structures and the appointment of the members of the certification commission were carried out according to plan by the executive committee of the DGHNO-KHC starting in November 2019. The certification commission then successfully developed the survey and key data sheet by July 2021. This comprises 35 items for the recording of structural, process, and outcome quality on the basis of the current CI CPG and the CI White Paper (Table 1).

**Table 1:** Excerpt from the survey and data sheet for recording the fulfillment of structural, process, and outcome quality

Requirement no	Requirement	Requirement fulfillment	
1	Is the White Paper used as the basis for the structure and work of your CI provision institution (CIVE)?	Yes	No
10	How many CI specialized audiologists according to the qualification profile, does the CIVE have?	Number:	
27	Are the required portions of the CI process provided by the CIVE? (# = no delegation, * = delegation possible)	– Presurgical evaluation (#) – Surgery (#) – Medical check-ups (#) – Audiological basic/follow-up therapy /aftercare (#) – Hearing rehabilitation basic/follow-up therapy /aftercare (*) – Speech therapeutic basic/follow-up therapy/aftercare (*) – Technical aftercare (*)	
33	Is the annual CI report/quality report published?	Yes	No
35	Is the CIVE’s participation in the CI registry guaranteed?	Yes	No

The complete questionnaire is available as a supplement (see Supplementary Information)

*CIVE* Cochlear Implant Provision Institution (*Cochlea-Implantat versorgende Einrichtung*)

At the beginning of 2021, the independent certification organization (the company ClarCert) was commissioned to implement a standardized certification process under the scientific direction of the DGHNO-KHC.

**Fig. 4:**
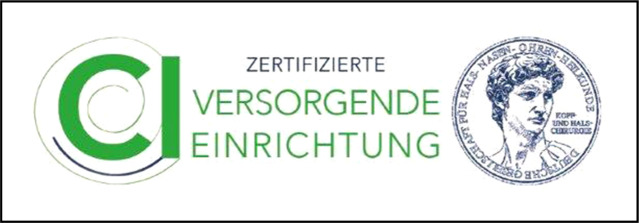
Display of the logo as evidence of successful certification as a “Cochlear Implant Provision Institution” (*Cochlea-Implantat versorgende Einrichtung*, CIVE) with authorization for internal and external use

After the establishment of a “Certificate Issuance Committee” and the creation of a certificate logo (Fig. 4), the certification procedure was formally initiated in August 2021 and announced via the DGHNO-KHC homepage as well as via a written mailing to the members of the DGHNO-KHC. As of September 2021, applications for the CIVE quality certificate could be submitted.

### Certification applications and certificate issuance

In the period from September to December 2021, 29 applications for CI certification were submitted by hospitals. In the period from January to December 2022, 22 applications were received. Thus, in the first 16 months since implementation of the certification process, 51 applications were submitted for the award of the certificate.

Of the applications received, 21 were approved in 2021 and 26 in 2022. This means that at the end of 2022, i.e., in the first 16 months (September 2021 to December 2022) since the certification process was implemented, a total of 47 certificates had been issued.

**Fig. 5:**
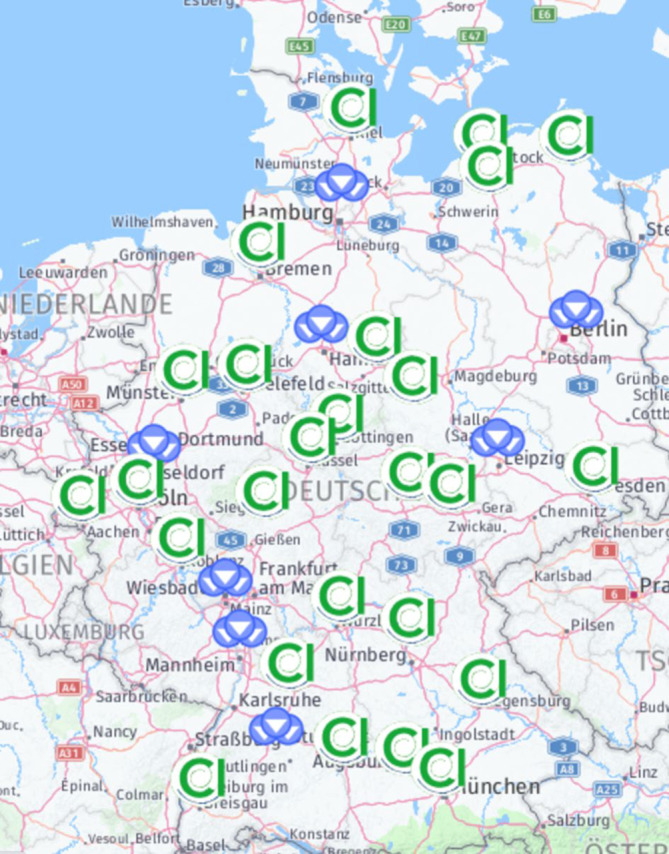
Graphical map representation of the currently successfully certified hospitals (Cochlear implant-provision institution, CIVE) in Germany. *Triangles *indicate locations with multiple CIVEs [7]

In 2021, it was not yet possible to issue a certificate for one application and in 2022 for three applications due to inconsistent information or non-compliance with the requirements. A list of successfully certified facilities is continuously compiled by the certification organization via its homepage [7]. This list is linked to the homepage of the DGHNO-KHC to make it publicly available. The presentation is in the form of a map with local marking of the CIVEs in Germany and in the form of a search mask (Fig. 5).

### Training of the technical experts

The training of the technical experts in preparation for the on-site auditing took place in March 2022. A total of 20 persons with the required basic qualification who had previously applied to participate in the training course took part in this training. After successfully completing the training course, 13 of the 20 participants were appointed as qualified technical experts or auditors to carry out the auditing.

### On-site auditing

After the first certificates were successfully awarded from October 2021, the first on-site audits by the trained technical experts took place as planned from July 2022. By the end of 2022, 13 on-site audits had already been carried out in practice. A further 27 on-site audits have been scheduled so far for 2023 to maintain the certificates. The on-site audits carried out to date showed a high level of agreement with the information previously provided by the hospitals on the basis of the survey and key data sheet, which was verified by the technical experts. In individual cases, recommendations were made for potential improvements, but these did not represent “critical deviations” and therefore did not stand in the way of certification to date (Table 2).

**Table 2:** Summary of the implementation of the certification program to date

	2021	2022	Total
Applications initial certification (off-site review)	29	22	51
Issued certificates (off-site review)	21	26	47
Certificates not eligible for decision or not issued (off-site review)	1	3	4
Completed on-site audit for initial certification	–	13	13

## Discussion

A reliable system has developed over the last 30 years, which can be considered innovative with regard to the development of the indication for CI therapy. Examples include bilateral surgery [8], CI for unilateral deafness [9, 10], and preservation of residual hearing with electro-acoustic stimulation [11], each of which has received significant impetus from Germany. Irrespective of an innovative environment, there are considerable differences regarding the care processes. This has resulted in a heterogeneous and non-standardized landscape for CI care in Germany. With the creation of the AWMF CI CPG and the CI White Paper, a uniform scientific consensus for CI care in Germany was successfully defined under the leadership of the DGHNO-KHC.

An independent review of the implementation of the CPG content has been almost impossible to date, e.g., for patients. Thus, on the one hand, clear recommendations for the structural, process, and outcome quality of CI care exist in Germany and, on the other hand, objective proof of compliance with the CPG recommendations was lacking. This gap should be closed by the introduction of the certification program of hospitals (CIVE) presented here.

The objective of the certification concept for CIVE is to implement the CI care process in accordance with the CI CPG and White Paper. In comparison, the certified “Audiological Centers” of the DGA [12] describe basic features of integrated diagnostics and care of complex hearing disorders, without exclusively listing the use of CIs or hearing implants. Although parallels can be found in the concepts in individual aspects, the two approaches show considerable differences in their objectives [13] and are therefore by no means to be regarded as equivalent.

The development of the organizational and structural foundations of the certification process already began in 2017, when fundamental decisions were made by the DGHNO-KHC to actively develop the topic of quality control of CI care on the basis of medical–scientific findings. After the consented third version of the CI CPG was published by the AWMF in October 2020, the CI White Paper could be created by the DGHNO-KHC in May 2021. With these documents, the essential cornerstones for the development of the certification program were available.

The certification program has been implemented very successfully on the basis of the concept and structure presented in this paper. Since December 2022, 47 hospitals for otorhinolaryngology have been certified as CIVE within just 1.5 years. The certificate and the use of the corresponding logo make it easy for patients and practitioners to recognize compliance with CPG-compliant structural, process, and outcome quality with regard to CI care. There is no question that this initiative is only at the beginning of its implementation and that a variety of challenges continue to exist, which will be the subject of future work and will be discussed in the sections that follow.

The number of 47 certified hospitals to date can be viewed and classified from different perspectives. With regard to the relative evaluation of this number, various factors must be taken into account, such as the population of Germany. This is currently approximately 84 million, so that, in purely mathematical terms, there is one certified facility for every 1.5 million residents. Whether this ratio is appropriate or corresponds to an oversupply or undersupply can neither be assessed nor evaluated on the basis of the figures collected. This is particularly true with regard to the international situation, for which there are hardly any reliable reference values against which it would currently be possible to orient oneself in a meaningful way.

The work presented here provides an initial indication of the number of facilities in Germany that follow the quality standards. It is worth considering in this context that the objective of the certification process was at no time to limit the number of hospitals offering CI care or to—indirectly—introduce a minimum quantity for care. Rather, the objective was exclusively to provide objective evidence of compliance with quality standards, in accordance with the applicable CI CPG and the CI White Paper. In this respect, the subject of this project is not the evaluation of whether 47 hospitals is a high or a low number for the care of the population in Germany.

Considering already existing surveys on the density of care in Germany, there is an earlier work addressing this issue. In the survey initiated by the DGHNO-KHC in 2020 [14], all 170 ENT hospitals in Germany existing at that time were asked about the provision of CI care. In this survey, 70 hospitals indicated that they offered CI therapy. However, the survey also indicated that the actual number of hospitals offering CI care was probably much higher, as less than half of the hospitals responded to the survey. The authors therefore assumed a number of approximately 100 hospitals. In relation to these data, the number of 47 certified hospitals so far seems quite low.

However, considering the very short period (approx. 1.5 years) of the current certification process, 47 successfully certified hospitals to date is a rather a high number. This great shows the widespread acceptance of the certification program by the hospitals and the high interest in participation. At present, we can only speculate about the reasons why individual hospitals have not yet decided to participate. Various factors, such as a lack of interest in the certificate, a lack of support from the hospital administration, an organizational delay on the part of the applicant, or a lack of financial or personnel resources, could be the reason for non-participation in the certification process. It is also possible, however, that applications are not currently being submitted by some facilities because they would recognizably fail to meet the applicable quality aspects of the CPG. In this scenario, a hospital could make a conscious decision not to submit a certification application at the moment, as it would likely be rejected. Since all applications received have now had a positive decision, have been rejected, or have to be additionally processed by the applicants, the implemented certification program can be considered fully functional and has had neither a positive nor a negative influence on the number of hospitals certified in the meantime.

A number of interesting aspects result from the numerous requirements that have to be fulfilled in terms of structural, process, and outcome quality by the applying facilities, and at the same time also the great interest of the hospitals to be able to prove a certified, high-quality CI care. During the preparation of individual certification applications, it was possible, not least due to the clearly defined structural requirements of a CIVE, to successfully argue with the respective hospital administration regarding additional equipment or requirements. This perception reflected back from the applicants can be seen as a constructive, positive effect of the certification process, since it was possible to close quality-relevant structural, equipment-related, or qualitative gaps in a hospital. In this respect, the introduction of the certification program for CI care is not only active proof of existing quality of a facility, but has already contributed to an actual improvement in the quality of care in some hospitals during this short period.

With regard to the use of a certification program for further quality-relevant developments, the continuous collection of clinically relevant data from patient treatment is indispensable. In order to further develop objective standards in the future, the establishment of clinical registries is of particular importance. Especially in the wake of the problem of defective breast implants [15], there is also a high level of interest in the introduction of clinical registries within health policy. The German *Implantateregistergesetz *(IRegG, Implant Registry Act; [16]) has already created the legal basis for this in Germany. In this law, besides other implants, the CI is also specifically named as a target of future registries. Taking this perspective into account, the DGHNO-KHC has worked out the prerequisites for the creation of a national CI registry (*Deutsches Cochlea-Implantat-Register*, DCIR) at an early stage, which is currently in the practical implementation phase (INNOFORCE, Ruggell, Liechtenstein). In order to be able to act in this respect in the future, one of the necessary prerequisites for granting a certificate to a hospital is the documented assurance of participation in the national CI registry. This step ensures that certification is only possible if a hospital also contributes clinical data to the CI registry. By contrast, exclusive participation in the CI registry is also possible without certification. However, non-participation in the CI registry automatically results in the loss of the CIVE certificate. This ensures the future development of the quality standards and, in addition, essential aspects of the outcome quality, including possible complications or undesirable developments of the care, are collected systematically and nationwide. This approach also allows potential evidence gaps in the development of clinical CPGs to be identified and filled on a scientific basis.

The certification program presented here and successfully introduced in Germany is based on the medical–scientific principles of the CI CPG. There is no question that further technical developments (e.g., remote fitting) or changes in the indication, changes in the rehabilitation process (e.g., self-fitting of patients), or also internet-based technical–medical procedures (e.g., remote check) will find their place in the care of CI patients in the future. It will therefore be the task of the DGHNO-KHC, with the collaboration of the participating professional societies, to record the current medical standard and to incorporate it into a future version of the CPG. In this respect, the certification program presented here represents a universal instrument for recording structure-, process-, and outcome-relevant aspects in the hospitals in the future. There is no question that this is a dynamic system that will undergo further changes and adjustments. Nevertheless, certification for transparent documentation will be indispensable and relevant even under future quality content that has changed.

The development of guidelines is not subject to uniform rules at the international level. Medical law is the responsibility of the respective country. The development of medical standards is usually the responsibility of the respective leading medical society. In the example of CI care, this is usually the respective ENT society of the country concerned. The prerequisite for the establishment of a quality control system with combined certification is the development of a CPG. Very few data exist to date on the worldwide dissemination and use of CPGs for CI treatment. In a recently published study, a very heterogeneous picture could be derived for Europe with regard to the establishment of CPGs and registries. At the time of the study, national CPGs for CI care existed in only 16 of 42 countries [17]. However, the establishment of national CPGs is a mandatory prerequisite for the meaningful establishment of quality control measures with the help of certification. In a realistic view, however, it must be stated that both the economic and the structural prerequisites for establishing quality control measures differ considerably in different countries, so that the certification approach described for Germany does not necessarily have to be a way of quality control that can be implemented in another country. Nevertheless, the methodological approach described here can provide an example for developing a country-specific approach.

A critical aspect is the current lack of funding for the CI certification program in Germany. The organizational structuring, development of the standards, and also the practical implementation were worked out on a voluntary basis by the DGHNO-KHC. The costs for participation in the certification process (several thousand Euros per year) are currently covered exclusively by the applicant institutions. Currently, financial compensation for CI treatment does not differentiate between certified and non-certified hospitals. A clear demand on health insurances must therefore be to compensate the demonstrable effort for quality control, including certification, in the interest of patients and hospitals. This is especially critical in light of the drastic reduction in reimbursement for a CI surgery (DRG: D01B) that has been implemented for 2023. Maintaining the quality of medical care requires processes and structures that are accompanied by an objectively measurable (financial) cost. Using the example of CI care, the certification process described here shows that not only the implementation of and compliance with medical quality standards, but also the independent verification of the same requires considerable effort and thus time and, above all, money. It is imperative that the necessary financial resources be made available to the hospitals by the health insurances. This applies to both the CI certification process and the CI registry.

## Practical conclusion

- The work presented here demonstrates that within approximately 1.5 years the conceptualization, structuring, and practical implementation of a certification program for quality control of cochlear implant (CI) care in Germany was implemented very successfully by the DGHNO-KHC.
- The quality certificate of “Cochlear implant-provision institution” (*Cochlea-Implantat versorgende Einrichtung, *CIVE) was developed.
- The high acceptance of the CIVE certification program proves the support and functionality of the presented program, so that the developed structure can possibly be exemplary for other countries or also other fields of medicine.

## Supplementary Information

Table 1 contains supplementary material: Survey and key data sheet for initial certification as cochlear implant (CI) provision institution (CIVE)

## Declarations

For this article no studies with human participants or animals were performed by any of the authors. All studies mentioned were in accordance with the ethical standards indicated in each case.
