# Supplementary material for: Conception and implementation of a certification system for quality control of cochlear implant treatment in Germany
Source: HNO. 2023 Jun 12;71(Suppl 1):73–81. doi: 10.1007/s00106-023-01306-w (PMC10409661; doi:10.1007/s00106-023-01306-w)
Supplement: Supplementary file 1 — Table 1 contains supplementary material: Survey and key data sheet for initial certification as cochlear implant (CI) provision institution (CIVE) [file 106_2023_1306_MOESM1_ESM.docx]

| **Survey and key data sheet for initial certification as “Cochlear implant (CI) provision institution (CIVE)”**  **in accordance with the requirements of the German Society of Otorhinolaryngology, Head and Neck Surgery (DGHNO-KHC)** | | | |
| --- | --- | --- | --- |
| **(English translation of the original German Version)** | | | |
|  |  |  |  |
| **Legend** |  |  |  |
|  | Input by institution optional |  |  |
|  | Input required by institution |  |  |
|  |  |  |  |
|  |  |  |  |
|  |  |  |  |
| **Master data** | |  |  |
| Reg. Nr. | CIVE- |  |  |
| Institutions name | |  |  |
| Street |  |  |  |
| House number | |  |  |
| ZIP |  |  |  |
| City |  |  |  |
| Head (medical) of hospital (physician) | |  |  |
| Application for cochlear implant provision in children? | |  |  |
|  |  |  |  |
|  |  |  |  |
| **Further descriptions to demonstrate the fulfillment of requirements** | | |  |
| **1. General aspects of CI care** | | |  |
| **Nr.** | **Chapter Whitepaper** | **Requirement** | **Requirement fulfillment** |
| 1 | --- | Is the Whitepaper used as the basis for the structure and work of your cochIear implant provision institution (CIVE)? |  |
| 2 | 1. | Is the responsibility for the overall CI process carried by the CIVE? |  |
| 3 | 1. | Are all sub-steps of the process (indication (preoperative evaluation), surgery, fitting (basic therapy), rehabilitation (follow-up therapy), aftercare responsibly offered by the CIVE? |  |
| 4 | -- | Does CI provision for adults take place at the CIVE? |  |
| 5 | -- | Does CI provision for children take place at the CIVE? |  |
|  |  |  |  |
| **2. Structural requirements** | |  |  |
| **Nr.** | **Chapter Whitepaper** | **Requirement** | **Requirement fulfillment** |
| 6 | 2.1 | Is an interdisciplinary team of experts / cooperation structure provided (according to Whitepaper chap. 2.1)? |  |
| 7 | 3.1.6 | Does a structured and documented interdisciplinary case conference take place? |  |
| 8 | 3.2.2 | Are consultative services consistently available for critical care, neurosurgery, neuroradiology, and pediatric anesthesiology and critical care? |  |
|  |  |  |  |
| **3. Staff: Minimum requirements** | |  |  |
| **Nr.** | **Chapter Whitepaper** | **Key figure** | **Requirement fulfillment** |
| 9 | 2.3 | How many ENT specialists who specialize in CI does the CIVE have? |  |
| 10 | 2.3 | How many CI specialized audiologists according to the qualification profile, does the CIVE have? |  |
| 11 | 2.3 | How many hearing technicians according to the qualification profile does the CIVE have? |  |
| 12 | 2.3 | How many MTA-F or audiology assistants does the CIVE have? |  |
| 13 | 2.3 | For CI in children: How many specialists in phoniatrics & pediatric audiology does the CIVE have? |  |
| 14 | 2.3 | How many therapists in the speech / language therapy professions does the CIVE have? |  |
|  |  |  |  |
| **4. Apparatuses and instruments requirements** | |  |  |
| **Nr.** | **Chapter Whitepaper** | **Requirement** | **Requirement fulfillment** |
| 15 | 2.5 | Does the CIVE have hardware and software to check conventional hearing aids? |  |
| 16 | 2.5 | Does the CIVE have hardware and software for fitting for at least 3 different CI implant systems? |  |
| 17 | 2.5 | Does the CIVE have measurement equipment for click- and frequency-specific ABR, ASSR, and CERA (DIN EN 60645-7:2010-08) (with the possibility of measurement under anesthesia/sedation for pediatric care)? |  |
| 18 | 2.5 | Does the CIVE have electrocochleography capability (with the ability to measure under anesthesia/sedation for pediatric care)? |  |
| 19 | 3.2.2 | Is radiological imaging available for intraoperative positional control of the CI? |  |
| 20 | 3.2.3 | Is cranial nerve monitoring / facial nerve monitoring available intraoperatively? |  |
| 21 | 3.2.3 | Is an eABR available intraoperatively? |  |
|  |  |  |  |
| **5. Minimum patient numbers** | |  |  |
| **Nr.** | **Chapter Whitepaper** | **Requirement** | **Requirement fulfillment** |
| 22 | 2.6 | Are at least 1000 routine audiometry examinations performed at the CIVE per year? |  |
| 23 | 2.6 | Are at least 100 special audiological examinations performed at the CIVE per year? (e.g. clarification of CI indication and pediatric audiological diagnostics in pediatric care). |  |
|  |  |  |  |
| **6. Prozessablauf** | |  |  |
| **Nr.** | **Chapter Whitepaper** | **Requirement** | **Requirement fulfillment** |
| 24 | 3.1.1 | Is the implementation of hearing aid optimization in the CI fitting guaranteed? |  |
| 25 | 3.2.2 | Are implants from at least 3 different manufacturers offered? |  |
| 26 | 3.2.4 | Is the CI fitting performed under full inpatient conditions? |  |
| 27 | 3.3 | Are the required portions of the CI process provided by the CIVE? |  |
| 28 | 3.3 | For adult patients, is basic hearing therapy (and speech therapy, if appropriate), follow-up therapy (rehabilitation), and aftercare initiated responsibly? |  |
| 29 | 3.3.1 | In children: Is and interdisciplinary, pedagogic, and hearing-language therapy rehabilitation (follow-up therapy) responsibly initiated for children? |  |
| 30 | 3.5 | Is annual follow-up provided by the CIVE? |  |
|  |  |  |  |
| **7. Quality assurance** | |  |  |
| **Nr.** | **Chapter Whitepaper** | **Requirement** | **Requirement fulfillment** |
| 31 | 2.7 | Are the tasks performed in compliance with the relevant regulations (MPBetreibV, MPSV, MPG, training, and re-certification of personnel)? |  |
| 32 | 2.7 | Is an annual CI report prepared? |  |
| 33 | 4.1 | Is the annual CI report / quality report published? |  |
| 34 | 4.1 | Does a CI database already exist in the CIVE? |  |
| 35 | 4.2 | Is the CIVE's participation in the CI registry guaranteed? |  |
